# Macro and Microelements Drive Diversity and Composition of Prokaryotic and Fungal Communities in Hypersaline Sediments and Saline–Alkaline Soils

**DOI:** 10.3389/fmicb.2018.00352

**Published:** 2018-02-27

**Authors:** Kaihui Liu, Xiaowei Ding, Xiaofei Tang, Jianjun Wang, Wenjun Li, Qingyun Yan, Zhenghua Liu

**Affiliations:** ^1^School of Biological Science and Engineering, Shaanxi University of Technology, Hanzhong, China; ^2^State Key Laboratory of Lake Science and Environment, Nanjing Institute of Geography and Limnology, Chinese Academy of Sciences, Nanjing, China; ^3^State Key Laboratory of Biocontrol and Guangdong Provincial Key Laboratory of Plant Resources, School of Life Sciences, Sun Yat-sen University, Guangzhou, China; ^4^Environmental Microbiome Research Center and School of Environmental Science and Engineering, Sun Yat-sen University, Guangzhou, China; ^5^School of Minerals Processing and Bioengineering, Central South University, Changsha, China

**Keywords:** prokaryotic and fungal community, soils and saline sediments, macroelement, microelement, high-throughput sequencing, variance partitioning analysis

## Abstract

Understanding the effects of environmental factors on microbial communities is critical for microbial ecology, but it remains challenging. In this study, we examined the diversity (alpha diversity) and community compositions (beta diversity) of prokaryotes and fungi in hypersaline sediments and salinized soils from northern China. Environmental variables were highly correlated, but they differed significantly between the sediments and saline soils. The compositions of prokaryotic and fungal communities in the hypersaline sediments were different from those in adjacent saline–alkaline soils, indicating a habitat-specific microbial distribution pattern. The macroelements (S, P, K, Mg, and Fe) and Ca were, respectively, correlated closely with the alpha diversity of prokaryotes and fungi, while the macronutrients (e.g., Na, S, P, and Ca) were correlated with the prokaryotic and fungal beta-diversity (*P* ≤ 0.05). And, the nine microelements (e.g., Al, Ba, Co, Hg, and Mn) and micronutrients (Ba, Cd, and Sr) individually shaped the alpha diversity of prokaryotes and fungi, while the six microelements (e.g., As, Ba, Cr, and Ge) and only the trace elements (Cr and Cu), respectively, influenced the beta diversity of prokaryotes and fungi (*P* < 0.05). Variation-partitioning analysis (VPA) showed that environmental variables jointly explained 55.49% and 32.27% of the total variation for the prokaryotic and fungal communities, respectively. Together, our findings demonstrate that the diversity and community composition of the prokaryotes and fungi were driven by different macro and microelements in saline habitats, and that geochemical elements could more widely regulate the diversity and community composition of prokaryotes than these of fungi.

## Introduction

Microbial communities are ubiquitous and can even thrive in extreme environments such as hypersaline lakes, marine habitats and saline–alkaline soils (Jiang et al., 2007; Andrei et al., 2015; Yakimov et al., 2015; Xie et al., 2017). They are key components of these extreme ecosystems, and thus play central roles in their geochemical cycles and ecological stability (Nunoura et al., 2013; Liang et al., 2014; Sorokin et al., 2015). Microbial communities in extreme environments have formed unique community structures (Oren, 2013). Moreover, the species diversity and distribution patterns of microbial assemblages vary, together with dynamic ecological variables such as pH, geochemical elements, moisture and terrestrial locations (Xiong et al., 2012; Liu K. et al., 2014; Bryanskaya et al., 2016; Fernandez et al., 2016; Zhong et al., 2016). Revealing the linkage between the diversity and structures of microbial communities and environmental driving forces will provide significant insight into the mechanisms that microbial communities use to adapt to extreme habitats, community succession and ecological functioning.

Accumulating evidence suggests that geochemical elements could be major factors influencing microbial assemblages (Oren, 2013). For example, macroelements such as K^+^, Ca^2+^, and Mg^2+^significantly contributed to the species richness and compositions of prokaryotic communities in soils and hypersaline sediments (Podell et al., 2014; Bryanskaya et al., 2016; Xia et al., 2016; Zhong et al., 2016) and Ca and P significantly affect fungal community compositions in forest soils (Sun et al., 2016). Although some microelements are regarded as toxic to most forms of life, they regulated microbial communities in extreme conditions. For instance, they are significantly correlated with bacterial community composition in hot springs (Jiang et al., 2016) and certain elements (Co, Ni, and Mn) explained variations of prokaryotic and fungal assemblages in polymetallic mining areas (Reith et al., 2015). These previous studies have enriched our knowledge of microbial biogeography, but the respective reports are mainly focused on various macroelements. In fact, microbial communities are jointly driven by various factors. What’s more, these two types of elements drive different physiological processes of microbial life (Stolz et al., 2006; Wintsche et al., 2016). Therefore, it is important to investigate the effects of these multiple variables on the diversity and composition of microbial communities. However, the relevant knowledge is still unavailable especially as regards saline habitats.

Salt lakes and saline–alkaline lands are widely distributed across the earth, where they are characterized by high osmotic pressure and high ionic concentrations (Oren, 2002; Hallsworth et al., 2007). Microbial communities in saline habitats are adapted to high ionic conditions, and salt ions play an important role in driving the diversity and composition of microbial communities. In this study, we collected 36 samples (including hypersaline sediments and saline soils) from salt lakes and saline–alkaline regions of northwest China. The diversity and composition of prokaryotic and fungal communities in these saline samples were determined based on the sequencing data of 16S rRNA genes and fungal ITS regions on an Illumina HiSeq. Specifically, this study has two major objectives: (1) evaluate the influence of macro- and microelements on the diversity of prokaryotic and fungal communities; (2) assess the relative importance of macro- and microelements in driving the distribution of prokaryotic and fungal communities.

## Materials and Methods

### Site Description and Sample Collection

**FIGURE 1:**
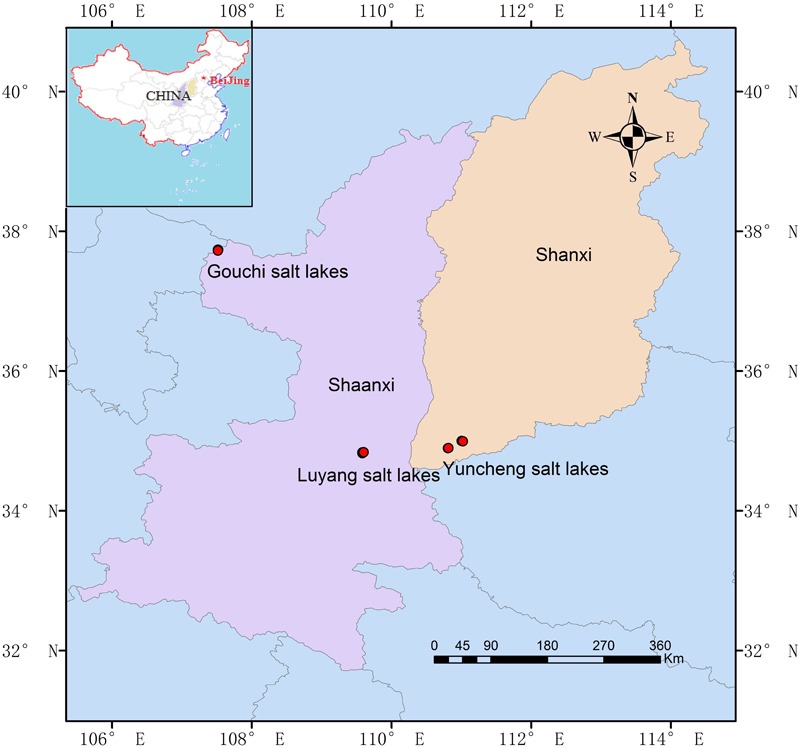
Geographic location of the Gouchi salt lake, Yuncheng salt lake, and Luyang salt lake. Location map of the sampling sites was created using ArcGIS software by Esri.

A total of 27 hypersaline sediment samples were collected from three salt lakes (Gouchi, Yuncheng, and Luyang lakes), and nine salinized soil samples were collected from areas adjacent to these lakes (**Figure 1**). Gouchi and Luyang lakes represent typical chloride-type lakes and are located in Dingbian County and Weinan City of Shaanxi Province in Northern China. Lake Yuncheng, known as one of the world’s three largest sodium-sulfate-type inland saline lakes, is located in Yuncheng City, in Shaanxi Province. Sediment samples from Gouchi, Yuncheng and Luyang lakes were numbered with the prefixes G, Y and W, respectively, and saline soil samples surrounding these three salt lakes were assigned the prefixes GT, YT and WT, respectively. Each sample was obtained by mixing five 10-g subsamples from 1 m × 1 m quadrants, which were taken from a depth of approximately 10–15 cm from a sediment or soil surface. Each set of five subsamples was combined as one sample, giving three biological replicates per plot. All samples were collected in sterile 50-ml plastic screw-top tubes, transported to the laboratory on ice, and stored at -80°C until the extraction of DNA.

### Measurements of Environmental Factors

Macroelements and trace elements in the soil and sediment samples were measured using an inductively coupled plasma mass spectrometer (ICP-MS; Perkin-Elmer Sciex Elan+ 5000). Briefly, sediment and soil samples were dried in a hot-air oven at 40°C until a stable weight was achieved, and sieved through a 0.25-μm filter. Each test sample (200 mg) was fully digested in a microwave digestion system with the addition of a mixture of HNO_3_ and HF (Arslan and Tyson, 2008), and the resulting solutions were transferred to 25-ml volumetric flasks and diluted to the fixed volume with 3% HNO_3_. The digestion procedure was performed in triplicate for each test sample. The calibration curve was prepared using a working standard solution with concentrations ranging from 0.01 to 10 μg ml^-1^ for all macroelements (Na, Mg, K, Ca, S, P, and Fe) and microelements (Al, As, B, Ba, Cd, Co, Cr, Cu, Ga, Ge, Hg, Li, Mn, Ni, Pt, Sr, Ti, Zn, and Rb). The concentrations of macroelements and trace elements in the soil and saline sediments were determined by ICP-MS (Sandroni and Smith, 2002). In addition, moisture was reported based on the water content of the samples. Geographic locations of each site were recorded in situ, and pH values of the samples were measured using pH strips with 1:5 (wt/vol) sediment to water.

### DNA Extraction, PCR Amplification, and Illumina-Based Sequencing

DNA was extracted from 0.5-g samples of soil or saline sediments using a FastDNA^TM^ SPIN Kit for soil (MP Biomedicals, LLC) according to the manufacturer’s instructions. Triplicate DNA extracts from the same sample were combined and analyzed using a NanoDrop^®^ TM ND-1000 spectrophotometer (Thermo Scientific, Wilmington, DE, United States). The 16S rRNA genes and the ITS regions of the extracted DNA were amplified using general bacterial primers 515F/806R (Caporaso et al., 2011) and fungal-specific primers ITS1F (Gardes and Bruns, 1993)/ITS4 (White et al., 1990). PCR was performed in triplicate for each sample with the following thermal cycling: an initial denaturation at 94°C for 5 min, followed by 25 cycles of 30 s at 94°C, 30 s at 50°C (ITS) or 55°C (16S rRNA) and 30 s at 72°C. Replicates were further pooled, and the resulting amplicons from each sample were sequenced on the HiSeq2500 system (Illumina Inc., San Diego, CA, United States).

### Sequence Processing

The quality filtering, chimera removal and operational taxonomic unit (OTU)-based clustering of the sequences were performed using the Mothur software V 1.35.1 (Quast et al., 2013). Briefly, sequences with a barcode ambiguity at one end and more than one ambiguous nucleotide (N), and those shorter than 200 bp in length after removal of the barcode and primer were discarded. Chimeric sequences were removed using the UCHIME modules in Mothur. The quality-filtered sequences were truncated to a constant length and were then clustered into OTUs with a 3% dissimilarity cutoff using the UPARSE pipeline (Edgar, 2013). The OTUs that contained fewer than two reads were excluded from further analysis. A representative sequence from each OTU was assigned to bacteria, archaea, and fungi using the Ribosomal Database Project (RDP) classifier (Wang et al., 2007). At the phylum-taxonomy level, OTUs in each of the three samples from the same saline pools or soil plots were combined and assigned, for example, (G1, G2, and G3) to G_1, (G4, G5, and G6) to G_2, (G7, G8, and G9) to G_3, (Y11, Y12, and Y13) to Y_1, (Y21, Y22, and Y23) to Y_2, (Y31, Y32, and Y33) to Y_3, (GT1, GT2, and GT3) to GT, (YT1, YT2, and YT3) to YT and (WT1, WT2, and WT3) to WT.

### Multivariate Statistical Analysis

All statistical analyses in this study were performed using the vegan package in R software, except where otherwise noted. Prior to data analysis, the number of sequences was normalized by randomly sub-sampling reads of prokaryotes (30,126 sequences) and fungi (30,198 sequences) for each sample to avoid a potential bias caused by different sequencing depths. The alpha and beta diversities were calculated using species-level OTUs in QIIME (Caporaso et al., 2010). Relationships of environmental variables and alpha-diversity indices (species richness and Shannon) with factors were tested based on Pearson’s correlation. Principal component analyses (PCoA), based on unweighted UniFrac metrics, were performed to reveal the spatial patterns of the microbial communities within the samples. Canonical correspondence analysis (CCA) was used to identify the significant factors (*P* ≤ 0.05) to the community composition of prokaryotes and fungi. The permutational multivariate analysis of variance (PerMANOVA) was performed using the distance matrices (R: adonis) in vegan with 999 permutations, and variation partitioning analyses (VPA) were performed by the vegan package in R 2.14.0 to evaluate the relative contribution of significant factors (*P* ≤ 0.05, variance inflation coefficient < 10) to the prokaryotic and fungal community structures. Due to the microbial community shaped by multiple factors, we also analyzed the joint effects of macroelements, microelements and other factors on the community composition.

### Accession Numbers

The raw sequencing data generated in this study were deposited into the National Center for Biotechnology Information (NCBI) database under accession numbers SAMN07764381-SAMN07764416 (16S rRNA) and SAMN07764417-SAMN07764452 (ITS).

## Results

### Characteristics of Environmental Variables

The macroelemental contents differed significantly among the 36 samples of salt-lake sediments and saline–alkaline soils (Supplementary Table S1). The Na content in all samples was variable. The concentrations of P, Ca, K, and Fe were low in samples G1, G2, and G3 compared to the other 33 samples, as expected. Moreover, the average P concentration in the saline sediments from Gouchi Lake (G4 to G9) was higher than that in the adjacent soils, whereas the average P concentrations in the hypersaline sediments from Yuncheng Lake and Luyang Lake were lower than those in the adjacent soils. In contrast, the average concentrations of S and Mg in the sediments from the salt lakes were significantly higher than in the neighboring saline soils.

As shown in Supplementary Table S1, the microelemental contents (Al, Co, Cr, Ga, Hg, Mn, Ni, Sr, and Ti) of the 33 samples were higher than those of G1, G2, and G3. The concentrations of Al and Ba fluctuated greatly among all samples. Moreover, the average Al content was found to be greater in the saline soils surrounding Yuncheng and Luyang lakes than in the sediments from those salt lakes. The Sr content varied significantly in saline sediments from each lake but was relatively constant in the soil samples. Mn and Ti concentrations were relatively constant in the all samples, except for samples G1, G2, and G3, in which they were exceptionally low. The contents of Co, Cr, Ga, Hg, and Ni were relatively constant in all samples. In general, moisture content in saline sediments was greater than in their corresponding surrounding soils.

**FIGURE 2:**
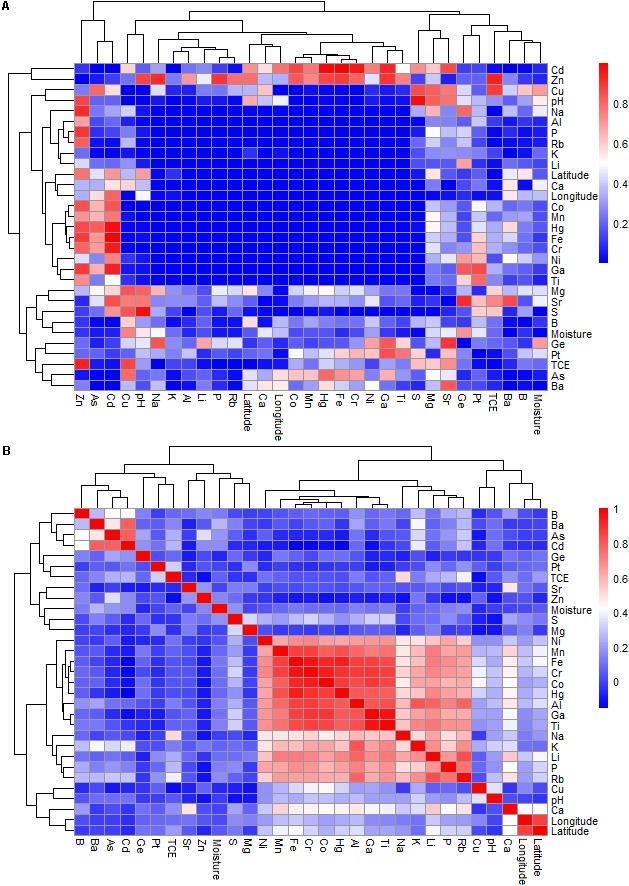
The Pearson’s correlation of geochemical variables of the study sites at the Gouchi salt lake, Yuncheng salt lake, and Luyang salt lake. **(A,B)** Represent *P*-value and correlation coefficient, respectively.

The Pearson’s correlations showed significant relationships between many environmental variables of the investigated sites (**Figure 2** and Supplementary Table S2). For example, the presence of S positively correlated with that of Cr (*P* = 0.005, *r* = 0.327) and Ga (*P* = 0.004, *r* = 0.310). The P content was significantly correlated with Na, K, Ca, Fe, Al, Co, Cr, Ga, Hg, Li, Mn, Ni, Rb, Ti, latitude and longitude, with *P* = 0.001 and *r* ranging from 0.213 to 0.740 for all correlations. The concentrations of Ca were correlated with Fe, Al, Co, Cr, Hg, Li, Mn, Rb, Sr, latitude and longitude (*P* = 0.001), with *r* values ranging from 0.420 to 0.541. The K content was correlated with Fe, Al, Co, Cr, Ga, Li, Mn, Ni, Rb and Ti, with *P* = 0.001 and *r* values of 0.491–0.809. The presence of Ba was positively related to Cd (*P* = 0.001 and *r* = 0.782). The Cr content had an even higher correlation to Na, Fe, Al, Co, Ga, Hg, Li, Mn, Ni, Rb, Ti, longitude and latitude (*P* = 0.001 and *r* values in the range of 0.258–0.959). The Ga content was correlated with Fe, Al, Co, Hg, Li, Mn, Ni, Rb and Ti at (*P* = 0.001 and *r* values of 0.616–0.959). The Ti content correlated with Na, Fe, Al, Co, Hg, Li, Mn, Ni, and Rb, with *P* = 0.001 and *r* values of 0.555 to 0.847. Moisture correlated with S (*P* = 0.015, *r* = 0.174) and Ba (*P* = 0.005, *r* = 0.256).

### Distribution Patterns of Microbial Communities

Illumina sequencing yielded 1,152,000 prokaryotic reads and 1,156,000 fungal sequences from the 36 samples, after removing low-quality tags and chimeras, resulting in a total of 43,579 OTUs and 3,182 OTUs, respectively (Supplementary Table S3). We found that the Chao1 index and observed OTUs for prokaryotes were significantly higher than these for fungi, and that the diversity of prokaryotes and fungi was higher in saline soils than in hypersaline samples (Supplementary Table S4). We normalized the sequence numbers to 30,124 and 30,198 reads per sample for prokaryotes and fungi, respectively, based on the fewest reads available among the 36 samples. These normalized reads were used in the subsequent analyses.

**FIGURE 3:**
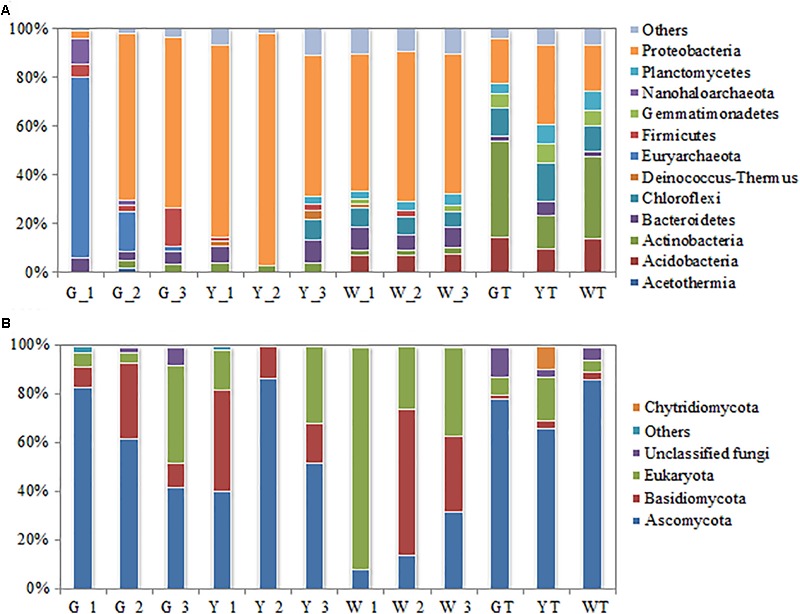
Relative abundance of the dominant phyla in the prokaryotic **(A)** and fungal **(B)** communities from the soil-saline sediment regions. Observed OTUs in three samples from the same saline pool (or soil plot) were combined and further assigned to G_1, G_2, G_3, Y_1, Y_2, Y_3, W_1, W_2, W_3, GT, YT and WT. Only the phyla with >1% mean abundance were shown.

The phylum-level distribution patterns of prokaryotes differed significantly between the hypersaline sediments and saline–alkaline soils (**Figure 3A**). A total of 74 prokaryotic phyla, including 13 abundant groups, were found across all samples. Proteobacteria were quite abundant in hypersaline sediments, accounting for 57–95% of the total reads per sediment sample (other than G_1, which was dominated by Euryarchaeota), while they exhibited lower richness in the soils. By contrast, Actinobacteria were the most abundant in saline soil samples (14–39%) but represented a relatively small proportion of prokaryotic reads in saline samples (<4%). This phylum was not detected in G_1. Bacteroidetes were more abundant in saline sediments than in soil samples, with the exception of sample Y_2, and Firmicutes were only present in some saline samples. However, Chloroflexi and Acidobacteria were richer in the soils than in the several salt-lake sediment samples.

Similarly, the community composition of fungi at the phylum level was distinct between the saline sediments and soils (**Figure 3B**). The relative abundances of Ascomycota varied greatly in the saline sediment samples (8–87%), while they were more consistent in the different soil samples (66–86%). Basidiomycota was more abundant in hypersaline sediments (9–60%) than in salinized soils (<3%), with the exception of sample W_1, which did not contain this group. Eukaryota inhabited all investigated sites but could not be assigned to any known group at the phylum level.

**FIGURE 4:**
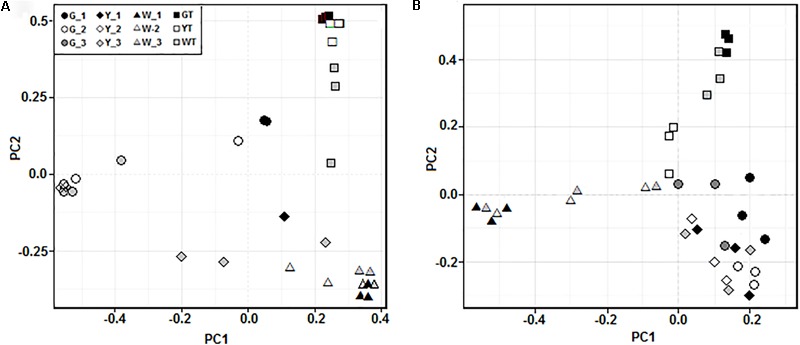
Principal coordinates analysis (PCoA) of the prokaryotic **(A)** and fungal **(B)** communities in all the samples along a salinity gradient. Microbial communities in three samples from the same saline pool (or soil plot) were represented by G_1, G_2, G_3, Y_1, Y_2, Y_3, W_1, W_2, W_3, GT, YT, and WT, respectively.

The PCoA analyses showed that prokaryotic (**Figure 4A**) and fungal (**Figure 4B**) communities in all samples were sorted by habitat, which was demonstrated by the community distributions at the phylum-level (**Figures 3A,B**). For example, the prokaryotic communities in hypersaline sediments were better separated along the PC1 axis, while these groups in saline soils were partly arrayed along the PC2 axis. The fungal communities in the sediments and saline soils exhibited the similar separation trend. Moreover, prokaryotic and fungal communities from the same saline pools or plots tended to be assembled together.

### Effects of Environmental Factors on Microbial Alpha Diversity

The rarefaction curves for all samples approached the saturation plateau (Supplementary Figure S1), indicating that the sequencing depths for each sample were sufficient to cover the microbial diversity. The species diversity of the prokaryotic community was higher in the soil samples than in the corresponding hypersaline sediments; the fungal community showed similar trends, with the exception of samples G3, Y13, and Y31 (Supplementary Table S4). In addition, species diversity of prokaryotic and fungal microbes was highly variable in the hypersaline sediment samples, with OTUs ranging from 74 to 2,394 and 12 to 366, respectively.

The Pearson’s analyses showed that the alpha-diversity of prokaryotic and fungal communities was influenced differently by macroelements (**Table 1**). The macroelements S (*P* = 0.001, *r* = 0.562 and 0.610, respectively), Mg (*P* = 0.002, *r* = 0.156 and 0.350, respectively) and Fe (*P* ≤ 0.047, *r* = 0.238 and 0.136, respectively) were significantly correlated with the Chao1 (species richness) and Shannon (diversity and evenness) indices of the prokaryotic community. Meanwhile, P (*P* = 0.021, *r* = 0.100) and K (*P* = 0.007, *r* = 0.118) were positively correlated with the Chao1 index of these groups. In contrast, Ca (*P* = 0.004, *r* = 0.228) correlated with the Shannon index of the fungal community.

**Table 1:** The Pearson correlations of alpha-diversity indices of prokaryotic and fungal communities with environmental factors.

Factor	Prokaryotic community (*P*/R)			Fungal community (*P*/R)	
	Chao1 (Richness)	Shannon		Chao1 (Richness)	Shannon
Na	0.489/-0.006	0.592/-0.033		0.280/0.042	0.067/0.156
S	**0.001/0.526**	**0.001/0.610**		0.320/0.012	0.319/0.029
P	**0.021/0.100**	0.214/0.049		0.067/0.181	0.188/0.080
Ca	0.223/0.026	0.085/0.088		0.690/-0.056	**0.004/0.228**
K	**0.007/0.118**	0.150/0.080		0.340/0.053	0.431/0.006
Mg	**0.002/0.156**	**0.002/0.350**		0.646/-0.075	0.158/0.103
Fe	**0.001/0.238**	**0.047/0.136**		0.494/-0.027	0.404/0.007
Al	**0.001/0.225**	**0.020/0.165**		0.347/0.029	0.356/0.023
As	0.878/-0.049	0.627/-0.042		0.143/0.146	0.776/-0.078
B	0.549/-0.012	0.269/0.033		0.299/0.031	0.784/-0.079
Ba	**0.001/0.287**	**0.012/0.196**		**0.001/0.467**	0.261/0.044
Cd	0.461/0.001	0.553/-0.030		**0.017/0.362**	0.514/-0.019
Co	**0.001/0.245**	**0.012/0.187**		0.414/0.013	0.491/-0.011
Cr	**0.001/0.295**	**0.011/0.201**		0.421/-0.012	0.455/-0.003
Cu	0.317/0.017	0.627/-0.037		0.591/-0.071	0.971/-0.156
Ga	**0.001/0.270**	**0.009/0.206**		0.342/0.008	0.215/0.064
Ge	0.200/0.033	0.072/0.144		0.322/-0.008	0.969/-0.125
Hg	**0.001/0.167**	0.159/0.072		0.417/-0.009	0.326/0.024
Li	0.259/0.022	0.475/-0.002		0.332/0.035	0.148/0.101
Mn	**0.001/0.218**	0.062/0.121		0.337/0.026	0.266/0.047
Ni	**0.004/0.167**	**0.025/0.158**		0.462/-0.021	0.216/0.072
Pt	0.918/-0.056	0.454/-0.025		0.524/-0.044	0.574/-0.047
Sr	0.417/0.004	0.177/0.064		0.712/-0.064	**0.001/0.433**
Ti	**0.001/0.282**	**0.009/0.225**		0.225/0.079	0.195/0.062
Zn	0.813/-0.041	0.931/-0.092		0.979/-0.117	0.837/-0.110
Rb	0.136/0.051	0.398/0.01		0.094/0.178	0.139/0.097
TCE	0.985/-0.076	0.899/-0.084		0.177/0.099	0.091/0.122
pH	0.779/-0.035	0.849/-0.07		0.396/0.015	0.390/0.016
Moisture	**0.004/0.158**	0.057/0.101		**0.005/0.275**	0.281/0.043
Longitude	**0.020/0.129**	**0.006/0.162**		0.930/-0.075	0.287/0.021
Latitude	**0.007/0.160**	**0.047/0.107**		0.836/-0.065	0.205/0.046

*Shannon: the Shannon index; Chao1: the Chao1 estimator. Significant *P* ≤ 0.05 are shown in bold. TCE represents the concentration of total elements.*

As shown in **Table 1**, the microelements had a greater effect on the species diversity of prokaryotic assemblages than on those of fungal communities. The microelements (Al, Ba, Co, Cr, Ga, Ni, and Ti) were significantly correlated with the Chao1 (*P* ≤ 0.004, *r* = 0.231 ± 0.064) and Shannon indices (*P* ≤ 0.025, *r* = 0.192 ± 0.033) of the prokaryotic community. Concentrations of Hg and Mn significantly influenced the Chao1 of prokaryotic taxa (*P* = 0.001, *r* = 0.167 and 0.218, respectively). However, for the fungal community, Ba and Cd were positively related to Chao1 (*P* ≤ 0.017, *r* = 0.467 and 0.362, respectively), and Sr was significantly correlated with Shannon index (*P* = 0.001, *r* = 0.433) as well.

Of other environmental variables, moisture was significantly correlated with the Chao1 index of the prokaryotic and fungal communities (*P* ≤ 0.005, *r* = 0.158 and 0.275, respectively). And, longitude and latitude were linked to the Chao1 (*P* ≤ 0.020, *r* = 0.129 and 0.160, respectively) and Shannon indices (*P* ≤ 0.047, *r* = 0.162 and 0.107, respectively) of the prokaryotic assemblages.

### Effects of Environmental Factors on Microbial Beta Diversity

**FIGURE 5:**
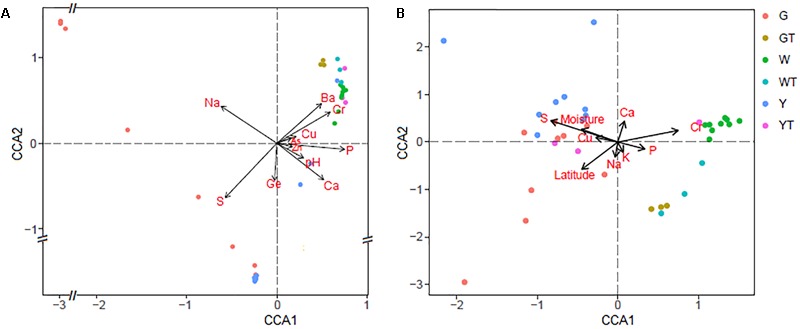
Canonical correspondence analysis (CCA) of the prokaryotic **(A)** and fungal **(B)** communities with the significant variables (*P* ≤ 0.05). G, Y, W, GT, YT, and WT represent microbial communities from Gouchi, Yuncheng, Luyang lakes, and the surrounding soils.

The CCA results showed significant correlations (*P* ≤ 0.05) between macro and microelements (e.g., Na, S, P, Ca, Ba, Cr, and Ge) and the prokaryotic microbial assemblages (**Figure 5A**), indicating that these geochemical parameters have strong impacts on the composition of the prokaryotic communities. In contrast, the CCA analysis also indicated that the trends of the factors, e.g., S, P, Ca, Cr, Cu, and moisture, correlated with the two axes and that these nutrient components significantly influenced the fungal community structures (*P* ≤ 0.05) (**Figure 5B**).

The PerMANOVA results indicated that the compositions of the prokaryotic communities in all samples were strongly driven by macroelements, including Na, S, P and Ca, with *P*-values ranging from 0.001 to 0.023 and *r*^2^-values ranging from 0.036 to 0.192. They were influenced by microelements As, Ba, Cr, Cu, Ge and Zn, with *P*-values varying from 0.001 to 0.048 and *r*^2^*-*values varying from 0.030 to 0.075. In addition, pH (*P* = 0.049, *r*^2^ = 0.031) correlated significantly with the compositions of the prokaryotic communities (**Table 2**). However, the fungal community structures were significantly influenced by the macroelements, including Na (*P* = 0.007, *r*^2^ = 0.041), S (*P* = 0.001, *r*^2^ = 0.074), P (*P* = 0.004, *r*^2^ = 0.043), Ca (*P* = 0.016, *r*^2^ = 0.036), and K (*P* = 0.003, *r*^2^ = 0.043), and by the microelements Cr (*P* = 0.006, *r*^2^ = 0.039) and Cu (*P* = 0.008, *r*^2^ = 0.039). Latitude shaped the compositions of fungal communities (*P* = 0.038, *r*^2^ = 0.033) (**Table 2**).

**Table 2:** The PerMANOVA of the community compositions of prokaryotes and fungi with geochemical factors.

Factor	*R*^2^	*P*
**Prokaryotic community**		
Na	0.092	**0.001**
S	0.191	**0.001**
P	0.047	**0.006**
Ca	0.036	**0.023**
Mg	0.007	0.846
As	0.040	**0.008**
B	0.019	0.251
Ba	0.031	**0.048**
Cr	0.036	**0.029**
Cu	0.030	**0.048**
Ge	0.034	**0.014**
Ni	0.017	0.277
Zn	0.075	**0.001**
pH	0.031	**0.049**
Moisture	0.031	0.066
**Fungal community**		
Na	0.041	**0.007**
S	0.074	**0.001**
P	0.043	**0.004**
Ca	0.036	**0.016**
K	0.043	**0.003**
Mg	0.027	0.117
Fe	0.029	0.088
Al	0.021	0.533
As	0.025	0.267
B	0.020	0.592
Ba	0.030	0.081
Cr	0.039	**0.006**
Cu	0.039	**0.008**
Ga	0.022	0.401
Ge	0.025	0.251
Hg	0.024	0.306
Li	0.021	0.534
Mn	0.027	0.171
Ni	0.030	0.073
Sr	0.026	0.194
Ti	0.026	0.169
Zn	0.021	0.531
Rb	0.026	0.170
TCE	0.020	0.593
pH	0.022	0.430
Moisture	0.032	0.051
Longitude	0.030	0.061
Latitude	0.033	**0.038**

*Significant *P* ≤ 0.05 are indicated in bold. TCE represents the concentration of total elements.*

### Determinants of the Microbial Community Composition

**FIGURE 6:**
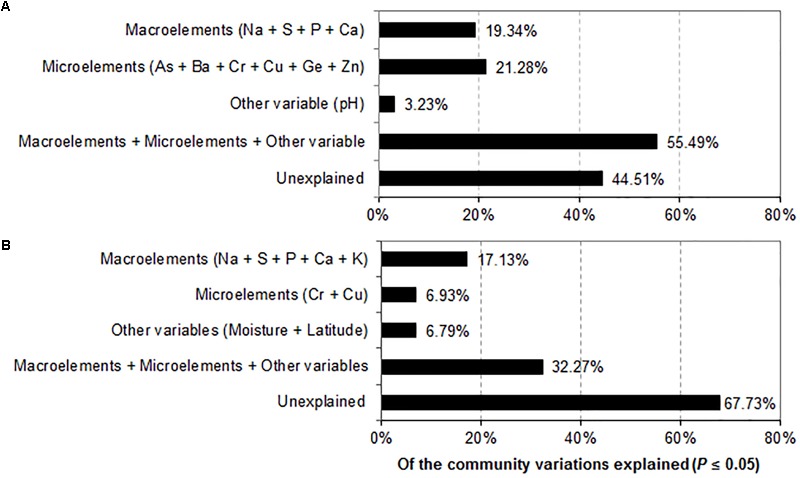
Variance partitioning analysis (VPA) demonstrating the effects of environmental factors on the variation of the prokaryotic **(A)** and fungal **(B)** community compositions.

Variance partitioning analyses were used to further quantify the relative effects of environmental factors on prokaryotic and fungal community structures. Macroelements (Na, S, P, and Ca), microelements (As, Ba, Cr, Cu, Ge and Zn) and pH contributed 19.34, 21.28, and 3.23% to the prokaryotic community variation, respectively. The combination of the significant geochemical variables (Na, S, P, Ca, As, Ba, Cr, Cu, Ge, Zn, and pH) (*P* ≤ 0.05) explained 55.49% of the prokaryotic community variation, leaving 44.51% of the variation unexplained (**Figure 6A**). In contrast, macronutrients (Na, S, P, Ca, and K), trace elements (Cr and Cu), other variables (latitude and moisture) accounted for 17.13%, 6.93% and 6.79% of the prokaryotic community compositions, respectively. The combination of the significant environmental factors (Na, S, P, Ca, K, C, Cu, moisture and latitude) (*P* ≤ 0.05) accounted for 32.27% of the observed variation in the fungal community, leaving 67.73% of the variation unexplained (**Figure 6B**).

## Discussion

In this study, we found that the average diversity index (Shannon index) of prokaryotes and fungi in the hypersaline sediments of salt lakes was consistently lower than in the adjacent saline–alkaline soils, and examined a noticeable decreasing trend in average species richness (Chao1 index) of the microbial communities in the hypersaline sediments (Supplementary Table S4), suggesting that the extreme saline environments harbor the lowest alpha-diversity of prokaryotes and fungi. It was agreed well with the general principle for ecology that microbial diversity is low in extreme habitats (Benlloch et al., 2002; Pavloudi et al., 2017). Furthermore, we found that the species richness and diversity of prokaryotes and fungi in the sediments drastically changed but remained relatively constant in the soils. This fluctuation of alpha-diversity indices might be caused by geochemical variations in the hypersaline sediments (Supplementary Table S1).

As expected, prokaryotic and fungal communities in hypersaline sediments and saline soils exhibited a habitat-dependent distribution pattern. Proteobacteria dominated hypersaline sediments (except for G_1), whereas many classes, such as Proteobacteria, Actinobacteria, Chloroflexi and Acidobacteria, were relatively evenly distributed in the saline soils (**Figure 3A**). Some groups of Proteobacteria widely occupied hypersaline habitats (Hollister et al., 2010; Quaiser et al., 2011; Kambura et al., 2016). Similarly, Ascomycota dominated the fungal communities in the soil samples, while its abundance varied obviously in the sediments. Basidiomycota were more abundant in the various hypersaline sediments and less in the soils (**Figure 3B**). Notably, prokaryotic and fungal communities were similar to each other when their habitats (hypersaline sediments versus saline soils) were similar, even if geographic distance between sample sites was greater, suggesting that habitat types play an important role in driving the microbial beta diversity.

The findings in this study demonstrate that the macroelements S, P, K, Mg, and Fe were drivers of the alpha-diversity of the prokaryotic communities in the salt-lake sediments and saline soils (*P* ≤ 0.05) (**Table 1**). Previous reports have mainly addressed that elements K^+^, S^2-^, and Mg^2+^ determine the species richness of archaeal communities in a hypersaline lake (Podell et al., 2014), and that K^+^, Mg^2+^, and total P affect the OTU richness of soil bacterial groups such as Chloroflexi, Actinobacteria, Proteobacteria, and Bacteroidetes (Kim et al., 2016; Xia et al., 2016). Aside from the macronutrients (S, P, K, and Mg) associated with the diversity, we also explored the importance of Fe driving the prokaryotic alpha diversity. Here, we only analyzed the correlation of Ca (*P* = 0.004) with the Shannon index of the fungi in saline habitats (**Table 1**). Ca as well was found to regulate global soil fungal diversity (Tedersoo et al., 2014). It might be because Ca^2+^-mediated signals pathways are indispensable for eukaryotes’ life processes including salt-stress tolerance (Shi et al., 2011; Asano et al., 2012; An et al., 2014). In contrast, K^+^ is crucial to the survival of halotolerant and halophilic prokaryotes in saline environments because these microbes concentrate K^+^ inside cells to maintain their abundance (Edbeib et al., 2016). This could partially explain why the alpha-diversity of prokaryotes and fungi in saline habitats are driven by different macroelements.

Studies have showed that the geochemical conditions of habitats govern microbial community compositions (Wang et al., 2013, 2017; Hazard et al., 2014; Oloo et al., 2016). Here, we observed that macroelements (Na, S, P, and Ca) shaped prokaryotic community compositions across the studied sites (*P* ≤ 0.05) (**Table 2**). These macroelements explained 19.34% of the total variation in prokaryotic community structures (**Figure 6A**). The significance of Na, S, and Ca concentrations for bacterial community structures has been suggested by research conducted on the mine tailings and salt lakes of the Tibetan Plateau (Valentín-Vargas et al., 2014; Zhong et al., 2016). We showed that Na, S, P, Ca, and K were significantly correlated with fungal community compositions (**Table 2**) and that these macronutrients explained 17.13% of the total variation (**Figure 6B**). S, P, and Ca were found to regulate fungal community compositions in a variety of soil habitats (Jie et al., 2012; Tedersoo et al., 2014), however, K and Na as drivers of fungal beta diversity were rarely reported in previous studies. Macroelements are responsible for multiple microbial processes, such as the synthesis of biological macromolecules, signal transduction and osmotic balance (Dominguez, 2004; Jung and Bahn, 2009; Madigan et al., 2009; Edbeib et al., 2016); thus, they play a crucial role in driving the community composition of prokaryotes and fungi.

We revealed a series of microelements (Al, Ba, Co, Cr, Ga, Hg, Mn, Ni, and Ti) influencing the alpha-diversity of prokaryotes (**Table 1**). As shown in previous studies, metal elements Al, Cr, Hg, Mn, and Ni shape the species richness of bacteria in soils or in marine sediments (Faoro et al., 2010; Liu Y.R. et al., 2014; Pereira et al., 2014; Quero et al., 2015; Zhang et al., 2015), but correlations between microchemicals (Ba, Co, Ga, and Ti) and prokaryotic alpha diversity had not been previously discerned. Although some microelements (e.g., Co, Cr, Hg, and Mn) are toxic to life, microchemicals with a suitable content serve as electron donors or acceptors and enzymatic activators of bacterial cells (Stolz et al., 2006; Huang et al., 2015; Wintsche et al., 2016). What’ more, it is known that diverse prokaryotes tolerate the effects of certain metals, and that microbial assemblages are significantly more resistant to heavy metals than pure cultures (Oregaard and Sørensen, 2007; Mejias Carpio et al., 2018). Therefore, microelements might be not toxic and instead be drivers of alpha-diversity of prokaryotic groups. Moreover, one study has shown that deep-sea hydrothermal sediments, rich in metal elements such as Ba and Sr, harbored the highest level of species diversity and phylogenetic uniqueness for eukaryotes (López-García et al., 2003), suggesting that some microelements could modulate fungal alpha-diversity. Here, our results showed that Ba correlated with the species richness of fungi (*P* = 0.001), while Sr correlated with the Shannon diversity, highlighting the importance of alkali-metal elements for the fungal alpha-diversity.

Similarly, microelements explained the variations in prokaryotic community composition (**Table 2**). For example, the elements As, Ba, Cr, Cu, Ge, and Zn significantly contributed to the differences in prokaryotic community structures in both hypersaline sediments and saline soils. Our findings are supported by research showing that microelements, including As, Cr, Cu, and Zn, affected bacterial community structures in coastal sediments and metal-rich soils (Quero et al., 2015; Reith et al., 2015). The combined microelements (As, Ba, Cr, Cu, Ge, and Zn) and variables (Cr and Cu) individually explained 21.28% and 6.93% of the total variation in community structures of prokaryotes and fungi (**Figure 6**), indicating the relative importance of micronutrients in modulating prokaryotic composition. Together, the alpha and beta-diversities of prokaryotes were more widely influenced by macro and microelements as compared to fungi, suggesting that geochemical element profiles could better predict the diversity and community compositions of prokaryotes.

Furthermore, we observed that variables including moisture, longitude and latitude had relationships with the species richness and community compositions of prokaryotes and fungi, supported by previously published accounts (Talley et al., 2002; Tedersoo et al., 2014; Ding et al., 2015; Shay et al., 2015). However, it should be noted that given terrestrial coordinates determine the specific geochemical characteristics, which could have direct effects on microbial diversity and community composition. Moreover, latitudes, on a broad scale, are related to temperature, which is a well-known controller of the primary productivities and nutrient uptake of cells (Fuhrman et al., 2008). The diversity and community compositions of prokaryotes and fungi were driven by different variables, suggesting that distinct mechanisms are involved in the maintenance and succession of these two communities.

## Conclusion

We found that the prokaryotic and fungal community compositions in hypersaline sediments and saline–alkaline soils exhibited habitat-dependent patterns. We explored the concept that the diversity and composition of prokaryotic and fungal communities were driven by different macro and microelements. We also found that the diversity and composition of prokaryotes were more widely influenced by geochemical elements than those of fungal communities. Geochemistry selects unique microbial communities and, conversely, microbes drive geochemical cycles. Further studies could focus on the functional microbial communities involved in geochemical recycling in hypersaline environments.

## Author Contributions

KL and XD collected the samples and designed the research. KL, XD, and XT produced the data. KL, XD, JW, WL, QY, and ZL performed the data analysis. KL and XD wrote the manuscript.

## Conflict of Interest Statement

The authors declare that the research was conducted in the absence of any commercial or financial relationships that could be construed as a potential conflict of interest.

## References

[B1] An B. Chen Y. Li B. Qin G. Tian S. (2014). Ca^2+^-CaM regulating viability of *Candida guilliermondii* under oxidative stress by acting on detergent resistant membrane proteins. *J. Proteomics* 109 38–49. 10.1016/j.jprot.2014.06.022 24998432

[B2] Andrei A. S. Robeson M. S. II Baricz A. Coman C. Muntean V. Ionescu A. (2015). Contrasting taxonomic stratification of microbial communities in two hypersaline meromictic lakes. *ISME J.* 9 2642–2656. 10.1038/ismej.2015.60 25932617 PMC4817630

[B3] Arslan Z. Tyson J. F. (2008). Determination of trace elements in siliceous samples by ICP-MS after precipitation of silicon as sodium fluorosilicate. *Mikrochim. Acta* 160 219–225. 10.1007/s00604-007-0809-9

[B4] Asano T. Hayashi N. Kobayashi M. Aoki N. Miyao A. Mitsuhara I. (2012). A rice calcium-dependent protein kinase OsCPK12 oppositely modulates salt-stress tolerance and blast disease resistance. *Plant J.* 69 26–36. 10.1111/j.1365-313X.2011.04766.x 21883553

[B5] Benlloch S. López-López A. Casamayor E. O. Øvreås L. Goddard V. Daae F. L. (2002). Prokaryotic genetic diversity throughout the salinity gradient of a coastal solar saltern. *Environ. Microbiol.* 4 349–360. 10.1046/j.1462-2920.2002.00306.x 12071980

[B6] Bryanskaya A. V. Malup T. K. Lazareva E. V. Taran O. P. Rozanov A. S. Efimov V. M. (2016). The role of environmental factors for the composition of microbial communities of saline lakes in the Novosibirsk region (Russia). *BMC Microbiol.* 16(Suppl. 1):4. 10.1186/s12866-015-0618-y 26822997 PMC4895280

[B7] Caporaso J. G. Kuczynski J. Stombaugh J. Bittinger K. Bushman F. D. Costello E. K. (2010). QIIME allows analysis of high-throughput community sequencing data. *Nat. Methods* 7 335–336. 10.1038/nmeth.f.303 20383131 PMC3156573

[B8] Caporaso J. G. Lauber C. L. Walters W. A. Berg-Lyons D. Lozupone C. A. Turnbaugh P. J. (2011). Global patterns of 16S rRNA diversity at a depth of millions of sequences per sample. *Proc. Natl. Acad. Sci. U.S.A.* 108 4516–4522. 10.1073/pnas.1000080107 20534432 PMC3063599

[B9] Ding X. Peng X. J. Jin B. S. Xiao M. Chen J. K. Li B. (2015). Spatial distribution of bacterial communities driven by multiple environmental factors in a beach wetland of the largest freshwater lake in China. *Front. Microbiol.* 6:129. 10.3389/fmicb.2015.00129 25767466 PMC4341555

[B10] Dominguez D. C. (2004). Calcium signalling in bacteria. *Mol. Microbiol.* 54 291–297. 10.1111/j.1365-2958.2004.04276.x 15469503

[B11] Edbeib M. F. Wahab R. A. Huyop F. (2016). Halophiles: biology, adaptation, and their role in decontamination of hypersaline environments. *World J. Microbiol. Biotechnol.* 32:135. 10.1007/s11274-016-2081-9 27344438

[B12] Edgar R. C. (2013). UPARSE: highly accurate OTU sequences from microbial amplicon reads. *Nat. Methods* 10 996–998. 10.1038/nmeth.2604 23955772

[B13] Faoro H. Alves A. C. Souza E. M. Rigo L. U. Cruz L. M. Al-Janabi S. M. (2010). Influence of soil characteristics on the diversity of bacteria in the Southern Brazilian Atlantic forest. *Appl. Environ. Microbiol.* 76 4744–4749. 10.1128/AEM.03025-09 20495051 PMC2901723

[B14] Fernandez A. B. Rasuk M. C. Visscher P. T. Contreras M. Novoa F. Poire D. G. (2016). Microbial diversity in sediment ecosystems (evaporites domes, microbial mats, and crusts) of hypersaline laguna Tebenquiche, Salar de Atacama, Chile. *Front. Microbiol.* 7:1284. 10.3389/fmicb.2016.01284 27597845 PMC4992683

[B15] Fuhrman J. A. Steele J. A. Hewson I. Schwalbach M. S. Brown M. V. Green J. L. (2008). A latitudinal diversity gradient in planktonic marine bacteria. *Proc. Natl. Acad. Sci. U.S.A.* 105 7774–7778. 10.1073/pnas.0803070105 18509059 PMC2409396

[B16] Gardes M. Bruns T. (1993). ITS primers with enhanced specificity for basidiomycetes–application to the identification of mycorrhizae and rusts. *Mol. Ecol.* 2 113–118. 10.1111/j.1365-294x.1993.tb00005.x 8180733

[B17] Hallsworth J. E. Yakimov M. M. Golyshin P. N. Gillion J. L. D’Auria G. de Lima Alves F. (2007). Limits of life in MgCl2-containing environments: chaotropicity defines the window. *Environ. Microbiol.* 9 801–813. 10.1111/j.1462-2920.2006.01212.x 17298378

[B18] Hazard C. Gosling P. Mitchell D. T. Doohan F. M. Bending G. D. (2014). Diversity of fungi associated with hair roots of ericaceous plants is affected by land use. *FEMS Microbiol. Ecol.* 87 586–600. 10.1111/1574-6941.12247 24741702

[B19] Hollister E. B. Engledow A. S. Hammett A. J. Provin T. L. Wilkinson H. H. Gentry T. J. (2010). Shifts in microbial community structure along an ecological gradient of hypersaline soils and sediments. *ISME J.* 4 829–838. 10.1038/ismej.2010.3 20130657

[B20] Huang L. Wang Q. Jiang L. Zhou P. Quan X. Logan B. E. (2015). Adaptively evolving bacterial communities for complete and selective reduction of Cr(VI), Cu(II), and Cd(II) in biocathode bioelectrochemical systems. *Environ. Sci. Technol.* 49 9914–9924. 10.1021/acs.est.5b00191 26175284

[B21] Jiang H. Dong H. Yu B. Liu X. Li Y. Ji S. (2007). Microbial response to salinity change in Lake Chaka, a hypersaline lake on Tibetan Plateau. *Environ. Microbiol.* 9 2603–2621. 10.1111/j.1462-2920.2007.01377.x 17803783

[B22] Jiang Z. Li P. Van Nostrand J. D. Zhang P. Zhou J. Wang Y. (2016). Microbial communities and arsenic biogeochemistry at the outflow of an alkaline sulfide-rich hot spring. *Sci. Rep.* 6:25262. 10.1038/srep25262 27126380 PMC4850476

[B23] Jie W. Cai B. Zhang Y. Li J. Ge J. (2012). The effect of sulfur on the composition of arbuscular mycorrhizal fungal communities during the pod-setting stage of different soybean cultivars. *Curr. Microbiol.* 65 500–506. 10.1007/s00284-012-0183-7 22797887

[B24] Jung K. W. Bahn Y. S. (2009). The stress-activated signaling (SAS) pathways of a human fungal pathogen, *Cryptococcus neoformans*. *Mycobiology* 37 161–170. 10.4489/MYCO.2009.37.3.161 23983528 PMC3749383

[B25] Kambura A. K. Mwirichia R. K. Kasili R. W. Karanja E. N. Makonde H. M. Boga H. I. (2016). Bacteria and Archaea diversity within the hot springs of Lake Magadi and little Magadi in Kenya. *BMC Microbiol.* 1:136. 10.1186/s12866-016-0748-x 27388368 PMC4936230

[B26] Kim J. M. Roh A. S. Choi S. C. Kim E. J. Choi M. T. Ahn B. K. (2016). Soil pH and electrical conductivity are key edaphic factors shaping bacterial communities of greenhouse soils in Korea. *J. Microbiol.* 54 838–845. 10.1007/s12275-016-6526-5 27888456

[B27] Liang Y. Zhao H. Zhang X. Zhou J. Li G. (2014). Contrasting microbial functional genes in two distinct saline-alkali and slightly acidic oil-contaminated sites. *Sci. Total Environ.* 487 272–278. 10.1016/j.scitotenv.2014.04.032 24784752

[B28] Liu K. Ding X. Wang H. F. Zhang X. Hozzein W. N. Wadaan M. A. (2014). Eukaryotic microbial communities in hypersaline soils and sediments from the alkaline hypersaline Huama Lake as revealed by 454 pyrosequencing. *Antonie Van Leeuwenhoek* 105 871–880. 10.1007/s10482-014-0141-4 24563154

[B29] Liu Y. R. Wang J. J. Zheng Y. M. Zhang L. M. He J. Z. (2014). Patterns of bacterial diversity along a long-term mercury-contaminated gradient in the paddy soils. *Microb. Ecol.* 68 575–583. 10.1007/s00248-014-0430-5 24827389

[B30] López-García P. Philippe H. Gail F. Moreira D. (2003). Autochthonous eukaryotic diversity in hydrothermal sediment and experimental microcolonizers at the Mid-Atlantic Ridge. *Proc. Natl. Acad. Sci. U.S.A.* 100 697–702. 10.1073/pnas.0235779100 12522264 PMC141059

[B31] Madigan M. T. Martinko J. M. Dunlap P. V. Clark D. P. (2009). “Nutrition and cultures of microorganisms,” in *Brock Biology of Microorganisms* 12th Edn (San Francisco, CA: Benjamin-Cummings Publishing Company) 108–140.

[B32] Mejias Carpio I. E. Ansari A. Rodrigues D. F. (2018). Relationship of biodiversity with heavy metal tolerance and sorption capacity: a meta-analysis approach. *Environ. Sci. Technol.* 52 184–194. 10.1021/acs.est.7b04131 29172474

[B33] Nunoura T. Nishizawa M. Kikuchi T. Tsubouchi T. Hirai M. Koide O. (2013). Molecular biological and isotopic biogeochemical prognoses of the nitrification-driven dynamic microbial nitrogen cycle in hadopelagic sediments. *Environ. Microbiol.* 15 3087–3107. 10.1111/1462-2920.12152 23718903

[B34] Oloo F. Valverde A. Quiroga M. V. Vikram S. Cowan D. Mataloni G. (2016). Habitat heterogeneity and connectivity shape microbial communities in South American peatlands. *Sci. Rep.* 6:25712. 10.1038/srep25712 27162086 PMC4861955

[B35] Oregaard G. Sørensen S. J. (2007). High diversity of bacterial mercuric reductase genes from surface and sub-surface floodplain soil (Oak Ridge. USA). *ISME J.* 1 453–467. 10.1038/ismej.2007.56 18043664

[B36] Oren A. (2002). Molecular ecology of extremely halophilic Archaea and Bacteria. *FEMS Microbiol. Ecol.* 39 1–7. 10.1111/j.1574-6941.2002.tb00900.x 19709178

[B37] Oren A. (2013). “Life at high salt concentrations,” in *The Prokaryotes–Prokaryotic Communities And Ecophysiology* 4th Edn eds Rosenberg E. DeLong E. F. Thompson F. Lory S. Stackebrandt E. (Berlin: Springer-Verlag) 421–440.

[B38] Pavloudi C. Kristoffersen J. B. Oulas A. De Troch M. Arvanitidis C. (2017). Sediment microbial taxonomic and functional diversity in a natural salinity gradient challenge Remane’s “species minimum” concept. *PeerJ* 5:e3687. 10.7717/peerj.3687 29043106 PMC5642246

[B39] Pereira L. B. Vicentini R. Ottoboni L. M. (2014). Changes in the bacterial community of soil from a neutral mine drainage channel. *PLoS One* 9:e96605. 10.1371/journal.pone.0096605 24796430 PMC4010462

[B40] Podell S. Emerson J. B. Jones C. M. Ugalde J. A. Welch S. Heidelberg K. B. (2014). Seasonal fluctuations in ionic concentrations drive microbial succession in a hypersaline lake community. *ISME J.* 8 979–990. 10.1038/ismej.2013.221 24335829 PMC3996697

[B41] Quaiser A. Zivanovic Y. Moreira D. López-García P. (2011). Comparative metagenomics of bathypelagic plankton and bottom sediment from the Sea of Marmara. *ISME J.* 5 285–304. 10.1038/ismej.2010.113 20668488 PMC3105693

[B42] Quast C. Pruesse E. Yilmaz P. Gerken J. Schweer T. Yarza P. (2013). The SILVA ribosomal RNA gene database project: improved data processing and web-based tools. *Nucleic Acids Res.* 41 590–596. 10.1093/nar/gks1219 23193283 PMC3531112

[B43] Quero G. M. Cassin D. Botter M. Perini L. Luna G. M. (2015). Patterns of benthic bacterial diversity in coastal areas contaminated by heavy metals, polycyclic aromatic hydrocarbons (PAHs) and polychlorinated biphenyls (PCBs). *Front. Microbiol.* 6:1053. 10.3389/fmicb.2015.01053 26528247 PMC4602156

[B44] Reith F. Zammit C. M. Pohrib R. Gregg A. L. Wakelin S. A. (2015). Geogenic factors as drivers of microbial community diversity in soils overlying polymetallic deposits. *Appl. Environ. Microbiol.* 81 7822–7832. 10.1128/AEM.01856-15 26341204 PMC4616943

[B45] Sandroni V. Smith C. M. M. (2002). Microwave digestion of sludge, soil and sediment samples for metal analysis by inductively coupled plasma-atomic emission spectrometry. *Anal. Chim. Acta* 468:335 10.1016/S0003-2670(02)00655-4

[B46] Shay P. E. Winder R. S. Trofymow J. A. (2015). Nutrient-cycling microbes in coastal Douglas-fir forests: regional-scale correlation between communities, in situ climate, and other factors. *Front. Microbiol.* 6:1097. 10.3389/fmicb.2015.01097 26500636 PMC4597117

[B47] Shi S. Chen W. Sun W. (2011). Comparative proteomic analysis of the Arabidopsis cbl1 mutant in response to salt stress. *Proteomics* 11 4712–4725. 10.1002/pmic.201100042 22002954

[B48] Sorokin D. Y. Banciu H. L. Muyzer G. (2015). Functional microbiology of soda lakes. *Curr. Opin. Microbiol.* 25 88–96. 10.1016/j.mib.2015.05.004 26025021

[B49] Stolz J. F. Basu P. Santini J. M. Oremland R. S. (2006). Arsenic and selenium in microbial metabolism. *Annu. Rev. Microbiol.* 60 107–130. 10.1146/annurev.micro.60.080805.142053 16704340

[B50] Sun H. Terhonen E. Kovalchuk A. Tuovila H. Chen H. Oghenekaro A. O. (2016). Dominant tree species and soil type affect the fungal community structure in a boreal peatland forest. *Appl. Environ. Microbiol.* 82 2632–2643. 10.1128/AEM.03858-15 26896139 PMC4836437

[B51] Talley S. M. Coley P. D. Kursar T. A. (2002). The effects of weather on fungal abundance and richness among 25 communities in the Intermountain West. *BMC Ecol.* 2:7. 10.1186/1472-6785-2-7 12079496 PMC117440

[B52] Tedersoo L. Bahram M. Põlme S. Kõljalg U. Yorou N. S. Wijesundera R. (2014). Fungal biogeography. Global diversity and geography of soil fungi. *Science* 346:1256688. 10.1126/science.1256688 25430773

[B53] Valentín-Vargas A. Root R. A. Neilson J. W. Chorover J. Maier R. M. (2014). Environmental factors influencing the structural dynamics of soil microbial communities during assisted phytostabilization of acid-generating mine tailings: a mesocosm experiment. *Sci. Total Environ.* 500–501, 314–324. 10.1016/j.scitotenv.2014.08.107 25237788 PMC4253589

[B54] Wang J. Shen J. Wu Y. Tu C. Soininen J. Stegen J. C. (2013). Phylogenetic beta diversity in bacterial assemblages across ecosystems: deterministic versus stochastic processes. *ISME J.* 7 1310–1321. 10.1038/ismej.2013.30 23446837 PMC3695296

[B55] Wang Q. Garrity G. M. Tiedje J. M. Cole J. R. (2007). Naive Bayesian classifier for rapid assignment of rRNA sequences into the new bacterial taxonomy. *Appl. Environ. Microbiol.* 73 5261–5267. 10.1128/AEM.00062-07 17586664 PMC1950982

[B56] Wang X. B. Lü X. T. Yao J. Wang Z. W. Deng Y. Cheng W. X. (2017). Habitat-specific patterns and drivers of bacterial β-diversity in China’s drylands. *ISME J.* 11 1345–1358. 10.1038/ismej.2017.11 28282041 PMC5437346

[B57] White T. J. Bruns T. Lee S. Taylor J. W. (1990). “Amplification and direct sequencing of fungal ribosomal RNA genes for phylogenetics,” in *PCR Protocols, a Guide to Methods and Applications* eds Innis M. A. Gelfand D. H. Sninsky J. J. White T. J. (New York, NY: Academic Press) 315–322.

[B58] Wintsche B. Glaser K. Sträuber H. Centler F. Liebetrau J. Harms H. (2016). Trace elements induce predominance among methanogenic activity in anaerobic digestion. *Front. Microbiol.* 7:2034. 10.3389/fmicb.2016.02034 28018337 PMC5160323

[B59] Xia Z. Bai E. Wang Q. Gao D. Zhou J. Jiang P. (2016). Biogeographic distribution patterns of bacteria in typical Chinese forest soils. *Front. Microbiol.* 7:1106. 10.3389/fmicb.2016.01106 27468285 PMC4942481

[B60] Xie K. Deng Y. Zhang S. Zhang W. Liu J. Xie Y. (2017). Prokaryotic community distribution along an ecological gradient of salinity in surface and subsurface saline soils. *Sci. Rep.* 7:13332. 10.1038/s41598-017-13608-5 29042583 PMC5645410

[B61] Xiong J. Liu Y. Lin X. Zhang H. Zeng J. Hou J. (2012). Geographic distance and pH drive bacterial distribution in alkaline lake sediments across Tibetan Plateau. *Environ. Microbiol.* 14 2457–2466. 10.1111/j.1462-2920.2012.02799.x 22676420 PMC3477592

[B62] Yakimov M. M. La Cono V. Spada G. L. Bortoluzzi G. Messina E. Smedile F. (2015). Microbial community of the deep-sea brine Lake Kryos seawater-brine interface is active below the chaotropicity limit of life as revealed by recovery of mRNA. *Environ. Microbiol.* 17 364–382. 10.1111/1462-2920.12587 25622758

[B63] Zhang D. C. Liu Y. X. Li X. Z. (2015). Characterization of bacterial diversity associated with deep sea ferromanganese nodules from the South China Sea. *J. Microbiol.* 53 598–605. 10.1007/s12275-015-5217-y 26310303

[B64] Zhong Z. P. Liu Y. Miao L. L. Wang F. Chu L. M. Wang J. L. (2016). Prokaryotic community structure driven by salinity and ionic concentrations in Plateau Lakes of the Tibetan Plateau. *Appl. Environ. Microbiol.* 82 1846–1858. 10.1128/AEM.03332-15 26746713 PMC4784034

