# Supplementary material for: Macro and Microelements Drive Diversity and Composition of Prokaryotic and Fungal Communities in Hypersaline Sediments and Saline–Alkaline Soils
Source: Front Microbiol. 2018 Feb 27;9:352. doi: 10.3389/fmicb.2018.00352 (PMC5835090; doi:10.3389/fmicb.2018.00352)
Supplement: Supplementary file 1 [file Data_Sheet_1.PDF]

Supplementary information

Macro and Microelements Drive Diversity and Composition of Prokaryotic and Fungal Communities in Hypersaline Sediments and Saline-Alkaline Soils

Kaihui Liu[1*], Xiaowei Ding[1], Xiaofei Tang[1], Jianjun Wang[2], Wenjun Li[3], Qingyun Yan[4], Zhenghua Liu[5]

[1]School of Biological Science and Engineering, Shaanxi University of Technology, Hanzhong City, Shaanxi, 723001, China

[2]State Key Laboratory of Lake Science and Environment, Nanjing Institute of Geography and Limnology, Chinese Academy of Sciences, Nanjing 210008, China

[3]State Key Laboratory of Biocontrol and Guangdong Provincial Key Laboratory of Plant Resources, School of Life Sciences, Sun Yat-Sen University, Guangzhou, China

[4]Environmental Microbiome Research Center and School of Environmental Science and Engineering, Sun Yat-sen University, Guangzhou 510006, China

[5]School of Minerals Processing and Bioengineering, Central South University, Changsha, 410083, China

*Corresponding authors:

Dr. Kaihui Liu

Telephone & Fax: +86-916-2641661

E-mail: Kaihhui168@hotmail.com

Dr. Xiaowei Ding

E-mail: dxw518@163.com

Running title: Elements drive the microbial communities

Fig. S1. The rarefaction curves of the observed prokaryotic (a) and fungal (b) OTUs.

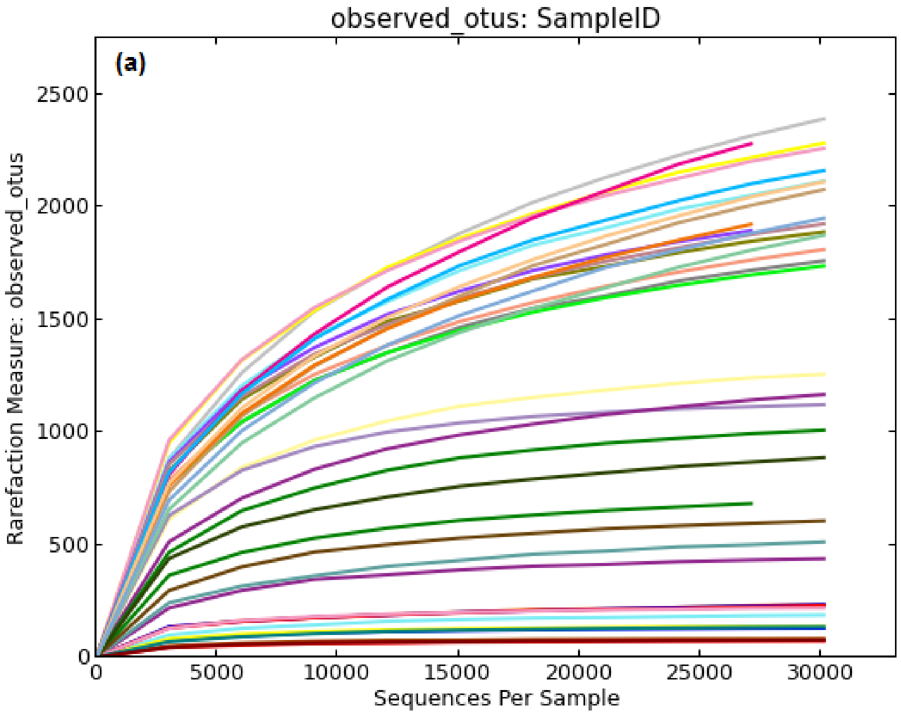

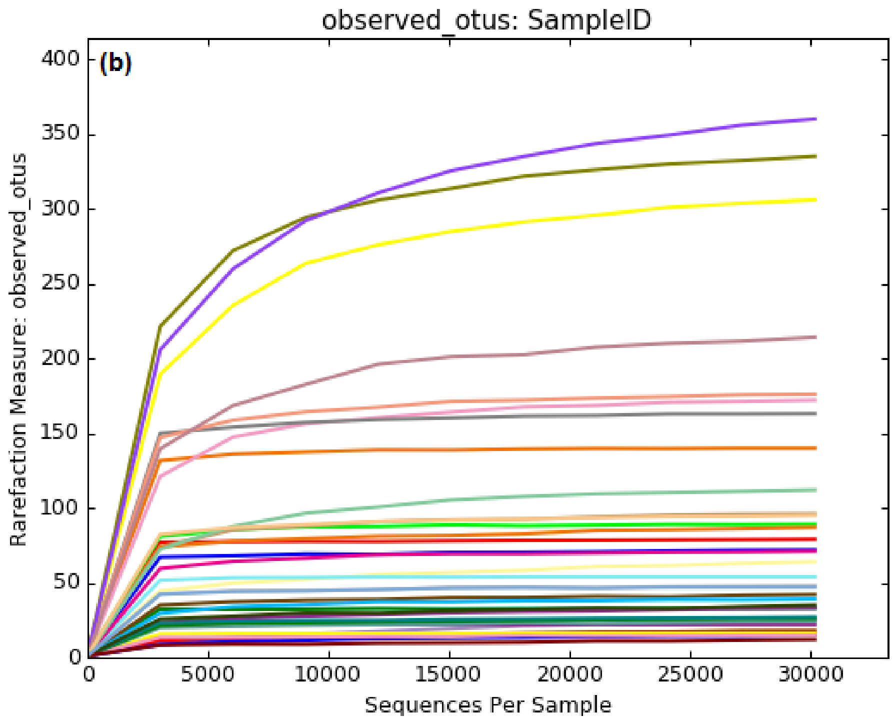

Table S1 Geochemical variables of the study sites examined by ICP-MS (mg $100g^{-1}$).

	Na	S	P	Ca	K	Mg	Fe	Al	As	B	Ba	Cd	Co	Cr	Cu	Ga	Ge	Hg	Li	Mn	Ni	Pt	Sr	Ti	Zn	Rb	TCE	pH	Longitude	Latitude	Moisture
G1	18768.758	4899.375	2.000	963.750	396.625	3173.125	87.250	107.750	3.750	27.250	6.375	0.000	0.000	0.750	0.250	0.875	0.000	0.000	1.000	2.375	4.750	0.625	13.750	5.375	8.750	4.750	28479.26	8.06	107.5174	37.7349	2.40
G2	38312.500	5243.125	2.250	226.875	299.500	1730.250	216.750	155.375	2.000	88.250	74.125	0.000	0.000	0.000	0.125	0.125	0.250	0.000	0.375	1.250	0.000	0.125	3.250	2.625	0.000	3.000	46362.13	7.94	107.5178	37.7346	1.22
G3	37500.000	1799.375	1.000	184.375	182.750	1208.125	152.125	56.625	0.250	36.750	7.000	0.000	0.000	0.125	0.000	0.125	0.125	0.000	0.250	0.625	0.000	0.125	3.250	2.125	5.500	3.125	41143.75	7.60	107.5180	37.7349	1.72
G4	6300.000	3350.000	17.625	4865.000	2217.500	1118.625	1424.375	4618.125	0.000	1183.750	8.625	0.000	1.250	3.875	1.125	18.750	0.000	9.375	4.250	39.125	1.750	0.250	55.750	244.000	12.125	16.750	25512.00	8.48	107.5186	37.7361	1.99
G5	11195.000	5567.500	32.875	4148.750	2440.625	1345.625	1416.875	4735.625	0.000	336.000	7.000	0.000	1.000	3.375	1.625	18.375	0.000	9.875	4.250	37.250	1.750	0.500	58.500	233.250	10.250	17.000	31622.88	8.40	107.5193	37.7358	1.78
G6	7233.125	4656.250	31.875	1984.375	1955.625	730.375	1175.125	4266.250	0.000	582.125	45.750	0.000	0.500	3.125	1.250	17.625	37.875	5.250	4.875	17.375	1.875	0.125	52.250	211.250	29.000	18.500	23061.75	8.45	107.5194	37.7355	1.55
G7	2155.250	3208.125	35.375	6650.000	2650.625	1415.000	2086.875	7193.750	3.750	753.375	261.000	0.000	1.500	4.875	1.375	22.000	7.875	12.000	5.000	36.125	1.750	0.250	57.375	237.625	17.750	19.625	26838.25	8.18	107.5157	37.7371	1.26
G8	5946.250	2001.250	47.250	6861.875	2676.875	1481.250	1810.625	6571.875	5.625	498.750	69.375	0.000	1.000	4.250	2.500	17.000	5.125	13.000	4.375	40.000	5.000	0.500	58.000	192.250	126.250	18.875	28459.13	8.42	107.5162	37.7370	1.60
G9	24694.375	2913.750	53.875	6534.375	2190.625	1908.125	1951.875	6243.125	0.000	604.250	32.375	0.000	1.250	4.875	1.875	15.750	2.000	11.625	5.000	45.375	4.125	1.250	73.625	219.500	17.000	18.500	47548.50	8.52	107.5167	37.7371	1.55
GT1	973.750	590.125	29.000	3070.000	2300.625	655.625	1610.000	6167.500	0.000	297.250	248.625	0.000	1.000	4.125	1.625	17.625	1.000	11.500	4.000	32.875	1.750	0.000	25.750	247.500	13.250	10.375	16314.88	8.08	107.5143	37.7229	0.38
GT2	1443.250	13.125	19.500	2385.625	2386.875	636.250	1646.250	5217.500	0.000	454.375	60.125	0.000	0.875	4.000	1.250	18.375	0.000	8.750	2.750	30.625	1.750	4.375	21.250	226.500	3.750	8.875	14596.00	8.49	107.5143	37.7225	0.44
GT3	1224.750	9.000	15.750	3356.250	2583.125	784.375	1898.125	6161.875	5.750	1570.625	607.875	0.250	1.375	4.750	1.250	25.000	3.875	10.125	5.125	34.500	1.750	0.125	27.125	281.000	18.375	13.625	18645.75	8.32	107.5141	37.7224	0.12
Y11	3016.875	3407.375	20.750	12493.750	1503.875	1810.000	1393.125	4418.125	0.000	2265.625	16.000	0.000	0.750	3.000	1.375	12.500	7.875	7.875	4.750	31.000	1.625	0.250	162.500	156.875	3.125	13.750	30752.75	8.37	111.0047	35.0004	1.41
Y12	4322.500	7773.750	30.125	13268.750	2073.125	2235.625	1619.500	6198.125	1.875	551.000	6.250	0.000	0.875	3.375	1.625	13.500	3.000	10.375	5.750	35.500	1.375	0.500	164.875	166.500	11.625	18.250	38517.75	8.23	111.0044	35.0002	1.31
Y13	3365.000	5703.125	38.375	10840.000	2679.375	2141.875	1873.750	7133.750	9.625	1515.000	51.625	0.125	1.000	4.125	1.875	19.625	6.250	11.250	5.875	44.500	2.000	0.250	183.625	236.250	375.250	21.750	36265.25	8.46	111.0041	35.0001	2.49
Y21	4760.000	4563.750	35.375	9860.000	1762.500	2934.375	1662.500	4713.125	0.000	311.250	12.875	0.000	0.875	3.500	1.750	12.000	0.000	9.250	5.875	40.125	1.375	1.125	137.625	143.000	10.375	16.000	30998.63	8.15	111.0230	34.9952	1.50
Y22	2521.875	4895.000	33.375	8200.000	1765.625	5135.000	2100.000	5794.375	0.000	247.375	15.375	0.000	1.125	4.500	2.375	16.625	0.000	12.750	6.750	45.000	1.625	1.125	132.625	209.500	6.125	17.875	31166.00	8.05	111.0229	34.9948	1.11
Y23	4237.500	4843.125	36.875	8906.250	2019.375	4522.500	2290.000	6015.000	0.000	292.375	11.875	0.000	1.125	5.000	2.500	17.125	0.000	15.375	7.125	49.375	1.875	1.375	133.000	222.625	4.250	18.000	33653.63	8.22	111.0230	34.9945	0.82
Y31	6785.000	2688.000	31.500	3896.875	4178.125	803.125	1891.250	8858.750	4.375	917.250	590.125	0.250	1.250	4.500	6.125	16.875	4.375	9.875	3.125	27.125	1.875	0.000	27.625	181.250	297.000	16.750	31242.38	8.33	110.8091	34.8968	1.07
Y32	3728.750	452.375	42.250	6250.625	2764.375	1429.375	2990.625	8390.000	0.000	433.500	165.250	0.000	1.750	6.875	8.875	28.750	0.000	18.625	6.125	48.500	3.500	1.125	44.000	328.875	69.125	18.000	27231.25	8.00	110.8080	34.8965	1.37
Y33	3739.375	400.000	43.375	5315.625	2892.500	1418.125	2909.375	9089.375	0.000	292.000	274.625	0.000	1.750	6.625	7.125	27.875	0.000	20.125	5.750	53.875	2.750	0.500	29.625	315.625	0.625	21.500	26868.13	8.77	110.8075	34.8964	1.60

YT1	7563.750	319.750	34.625	10835.000	3141.875	1343.125	2241.875	8613.125	5.250	790.500	673.875	0.250	1.375	6.125	2.625	19.625	4.625	12.375	5.750	64.125	2.750	0.125	42.750	227.500	7.875	23.625	35984.25	7.82	110.8152	34.8978	0.57
YT2	5591.250	513.125	46.750	8129.375	3375.625	1538.125	3012.500	9095.000	0.000	784.500	406.000	0.000	2.000	7.500	11.000	28.375	0.000	17.375	6.375	75.750	3.250	0.875	37.250	358.375	9.625	23.250	33073.25	7.64	110.8148	34.8976	0.48
YT3	5291.875	469.625	60.250	5831.875	2676.250	1589.375	3115.625	8133.750	0.000	159.500	156.250	0.000	2.000	7.625	10.750	27.500	0.000	20.625	5.375	62.250	3.375	1.375	25.625	325.375	62.000	20.500	28058.75	7.81	110.8149	34.8972	0.83
W11	3696.250	330.750	54.125	6670.625	2070.625	1202.000	2518.750	7521.875	0.000	652.000	43.625	0.000	1.250	5.875	2.000	26.500	0.000	14.375	5.500	47.000	2.500	0.750	107.125	313.000	16.625	19.750	25322.88	8.20	109.5860	34.8277	0.45
W12	3830.000	444.875	49.000	8342.500	2145.625	1184.250	2159.375	7106.875	0.000	671.125	299.250	0.000	1.125	5.250	1.625	19.875	0.000	12.750	5.500	44.125	2.000	0.375	154.000	252.250	64.875	19.875	26816.50	8.25	109.5856	34.8275	1.70
W13	8886.875	391.500	45.250	6216.250	2175.000	1086.125	2355.625	7188.125	0.000	604.750	158.125	0.000	1.375	5.500	1.875	24.625	0.000	13.125	5.250	49.750	2.375	0.625	50.250	308.125	12.625	18.250	29601.38	8.42	109.5855	34.8274	1.89
W21	4859.375	308.375	49.250	8070.625	1954.375	1486.875	2513.750	7493.750	0.000	292.125	41.375	0.000	1.250	5.375	2.125	18.125	0.000	15.750	5.875	63.250	3.000	0.625	71.500	229.625	15.000	21.375	27522.75	8.86	109.5852	34.8271	0.68
W22	6696.875	318.125	47.625	10563.125	2191.250	1309.625	2206.250	7145.625	0.000	259.500	222.875	0.000	1.250	5.250	1.875	22.875	0.000	11.125	5.625	52.875	2.500	0.375	260.250	289.750	91.500	20.750	31726.88	8.43	109.5847	34.8264	1.39
W23	6888.750	404.875	45.000	7630.000	2252.500	1142.875	2593.750	6866.250	0.000	707.250	182.375	0.000	1.500	5.875	2.250	25.875	0.000	16.625	5.250	56.875	2.625	0.625	61.500	296.500	43.375	18.750	29251.25	8.06	109.5843	34.8361	1.19
W31	11648.125	351.500	46.375	8657.500	2291.875	1404.250	2704.375	7442.500	0.000	230.250	202.750	0.000	1.500	6.125	2.250	22.750	0.000	18.000	6.125	61.750	2.750	0.625	113.000	310.000	19.500	21.375	35565.25	8.37	109.5831	34.8256	1.27
W32	16440.625	329.875	44.875	8743.750	2463.125	1411.875	2505.000	7784.375	0.000	567.000	343.625	0.000	1.500	5.750	2.125	25.000	0.000	16.625	5.750	53.500	2.625	0.375	106.750	311.000	32.625	20.875	41218.63	8.57	109.5831	34.8255	0.14
W33	17315.000	365.875	44.375	9013.125	2290.625	1446.250	2595.625	7315.000	0.000	261.750	240.875	0.000	1.500	6.000	2.250	24.750	0.000	15.375	6.250	59.625	3.000	0.500	151.250	300.750	50.125	22.500	41532.38	8.31	109.5830	34.8253	1.29
WT1	12630.000	218.000	48.750	6656.875	2730.625	1246.500	2692.500	8308.750	0.125	909.250	524.125	0.125	1.625	5.875	2.500	27.625	1.625	19.250	7.125	56.375	2.875	0.250	40.250	359.000	10.375	26.250	36526.63	8.33	109.5962	34.8365	0.10
WT2	17223.750	57.750	70.625	5396.875	2694.375	1380.000	2897.500	8434.375	0.000	387.250	345.500	0.000	1.750	6.500	2.625	28.625	0.000	17.375	6.625	64.000	3.250	0.750	35.875	367.125	15.875	22.250	39460.63	8.25	109.5980	34.8369	0.24
WT3	20786.250	78.500	71.125	9016.250	4241.875	996.500	2369.375	10216.875	15.250	1723.000	776.875	0.375	1.500	5.875	2.125	24.125	13.000	13.125	7.750	49.875	3.250	-0.375	43.625	354.500	14.750	33.500	50858.88	8.79	109.6009	34.8376	0.34

Note: TCE represents the concentration of total elements.

Table S2 The Pearson's correlation matrix between site geochemical properties.

		Na	S	P	Ca	K	Mg	Fe	Al	As	B	Ba	Cd	Co	Cr	Cu	Ga	Ge	Hg	Li	Mn	Ni	Pt	Sr	Ti	Zn	Rb	TCE	pH	Longitude	Latitude
S	*r*	0.012																													
	P	0.335																													
P	*r*	0.495	0.157																												
	P	**0.001**	**0.051**																												
Ca	*r*	0.303	0.222	0.415																											
	P	**0.008**	**0.005**	**0.001**																											
K	*r*	0.513	0.095	0.533	0.343																										
	P	**0.001**	0.160	**0.001**	**0.004**																										
Mg	*r*	-0.067	0.337	-0.021	0.066	0.062																									
	P	0.645	**0.004**	0.464	0.264	0.288																									
Fe	*r*	0.486	0.275	0.682	0.467	0.567	-0.015																								
	P	**0.001**	**0.006**	**0.001**	**0.001**	**0.001**	0.403																								
Al	*r*	0.574	0.242	0.707	0.472	0.809	0.045	0.856																							
	P	**0.001**	**0.019**	**0.001**	**0.001**	**0.001**	0.262	**0.001**																							
As	*r*	0.050	0.052	0.181	0.026	0.339	-0.043	-0.075	0.100																						
	P	0.237	0.189	0.087	0.336	**0.024**	0.502	0.709	0.160																						
B	*r*	0.077	0.015	0.231	0.201	0.274	-0.017	0.061	0.171	0.432																					
	P	0.214	0.350	**0.031**	**0.035**	**0.028**	0.399	0.224	0.087	**0.011**																					
Ba	*r*	-0.004	0.133	0.140	-0.027	0.400	-0.002	-0.004	0.216	0.468	0.270																				
	P	0.371	0.071	0.085	0.545	**0.004**	0.376	0.406	**0.038**	**0.004**	**0.029**																				
Cd	*r*	0.015	-0.063	0.143	-0.032	0.384	-0.066	-0.156	0.081	0.789	0.426	0.782																			
	P	0.312	0.726	0.128	0.560	**0.009**	0.606	0.998	0.229	**0.001**	**0.013**	**0.001**																			
Co	*r*	0.467	0.257	0.590	0.428	0.630	0.003	0.912	0.834	-0.060	0.071	0.075	-0.118																		
	P	**0.002**	**0.005**	**0.001**	**0.001**	**0.001**	0.380	**0.001**	**0.001**	0.643	0.235	0.207	0.834																		

Cr	*r*	0.525	0.327	0.659	0.456	0.619	-0.018	0.959	0.863	-0.069	0.077	0.047	-0.136	0.936												
	P	**0.001**	**0.005**	**0.001**	**0.001**	**0.001**	0.446	**0.001**	**0.001**	0.696	0.204	0.287	0.945	**0.001**												
Cu	*r*	-0.024	-0.080	0.081	-0.038	0.232	-0.115	0.337	0.212	-0.106	-0.064	0.016	-0.066	0.434	0.388											
	P	0.435	0.849	0.231	0.610	0.071	0.818	**0.017**	0.062	0.793	0.619	0.400	0.561	**0.001**	**0.007**											
Ga	*r*	0.548	0.310	0.613	0.404	0.641	0.063	0.855	0.808	-0.061	0.086	0.060	-0.122	0.874	0.876	0.224										
	P	**0.002**	**0.004**	**0.001**	**0.002**	**0.001**	0.231	**0.001**	**0.001**	0.650	0.186	0.222	0.935	**0.001**	**0.001**	**0.046**										
Ge	*r*	-0.086	0.086	-0.018	0.101	0.020	-0.018	0.064	0.036	0.217	0.139	0.068	0.176	0.090	0.013	-0.034	-0.086									
	P	0.834	0.168	0.426	0.190	0.272	0.368	0.179	0.203	0.061	0.103	0.176	0.128	0.185	0.300	0.410	0.825									
Hg	*r*	0.503	0.179	0.635	0.448	0.552	-0.030	0.935	0.799	-0.097	0.056	-0.033	-0.150	0.868	0.885	0.318	0.803	0.091								
	P	**0.003**	**0.024**	**0.001**	**0.001**	**0.002**	0.512	**0.001**	**0.001**	0.812	0.249	0.576	0.975	**0.001**	**0.001**	**0.014**	**0.001**	0.152								
Li	*r*	0.619	0.093	0.658	0.541	0.683	0.131	0.715	0.776	0.099	0.102	0.068	0.059	0.637	0.689	0.063	0.682	-0.075	0.695							
	P	**0.001**	0.157	**0.001**	**0.001**	**0.001**	0.166	**0.001**	**0.001**	0.211	0.178	0.218	0.209	**0.001**	**0.001**	0.287	**0.001**	0.685	**0.001**							
Mn	*r*	0.470	0.199	0.630	0.515	0.577	-0.029	0.860	0.765	-0.058	0.033	0.041	-0.090	0.789	0.850	0.257	0.712	0.116	0.820	0.716						
	P	**0.001**	**0.018**	**0.001**	**0.001**	**0.001**	0.514	**0.001**	**0.001**	0.645	0.325	0.310	0.760	**0.001**	**0.001**	**0.037**	**0.001**	0.138	**0.001**	**0.001**						
Ni	*r*	0.470	0.241	0.624	0.300	0.491	0.018	0.679	0.647	0.053	0.003	-0.019	-0.096	0.640	0.695	0.173	0.654	-0.071	0.625	0.555	0.625					
	P	**0.001**	**0.010**	**0.001**	**0.002**	**0.001**	0.344	**0.001**	**0.001**	0.269	0.404	0.492	0.739	**0.001**	**0.001**	0.101	**0.001**	0.689	**0.001**	**0.001**	**0.001**					
Pt	*r*	-0.007	-0.043	0.085	0.060	0.019	0.160	-0.048	-0.013	0.052	-0.012	0.059	0.104	-0.035	-0.055	0.054	-0.093	0.019	-0.022	0.164	-0.009	-0.060				
	P	0.333	0.586	0.177	0.227	0.256	0.088	0.594	0.387	0.199	0.384	0.159	0.129	0.507	0.646	0.192	0.839	0.270	0.444	0.090	0.378	0.616				
Sr	*r*	0.031	0.123	0.009	0.470	0.043	0.124	0.014	0.031	-0.015	0.096	-0.078	-0.096	0.036	0.036	-0.078	0.066	-0.097	0.015	0.081	0.020	-0.009	-0.055			
	P	0.263	0.070	0.372	**0.001**	0.273	0.107	0.342	0.272	0.445	0.162	0.849	0.845	0.242	0.283	0.758	0.162	0.915	0.351	0.182	0.302	0.454	0.629			
Ti	*r*	0.555	0.317	0.667	0.397	0.689	0.052	0.835	0.819	0.030	0.111	0.120	-0.036	0.847	0.858	0.177	0.959	-0.054	0.789	0.726	0.740	0.670	-0.083	0.057		
	P	**0.001**	**0.003**	**0.001**	**0.001**	**0.001**	0.240	**0.001**	**0.001**	0.241	0.164	0.121	0.507	**0.001**	**0.001**	0.099	**0.001**	0.589	**0.001**	**0.001**	**0.001**	**0.001**	0.795	0.220		
Zn	*r*	-0.112	0.094	-0.121	0.021	0.116	-0.026	-0.106	-0.077	0.348	0.150	0.042	0.167	-0.111	-0.095	0.064	-0.106	0.017	-0.109	-0.030	-0.080	-0.033	0.030	0.185	-0.084	
	P	0.914	0.140	0.889	0.368	0.241	0.397	0.881	0.700	0.057	0.117	0.268	0.122	0.821	0.802	0.229	0.893	0.199	0.863	0.463	0.730	0.440	0.195	0.065	0.741	
Rb	*r*	0.576	0.064	0.740	0.483	0.687	-0.005	0.631	0.776	0.298	0.265	0.270	0.294	0.607	0.635	0.028	0.616	-0.029	0.603	0.818	0.652	0.527	0.174	0.040	0.671	-0.111
	P	**0.001**	0.205	**0.001**	**0.001**	**0.001**	0.405	**0.001**	**0.001**	**0.031**	**0.038**	**0.021**	**0.027**	**0.001**	**0.001**	0.363	**0.001**	0.389	**0.001**	**0.001**	**0.001**	**0.001**	0.136	0.267	**0.001**	0.809

TCE	*r*	0.514	-0.033	0.264	0.169	0.123	-0.050	0.029	0.125	0.238	0.076	0.146	0.254	0.034	0.040	-0.127	0.040	0.075	0.026	0.233	0.071	0.100	0.316	-0.061	0.064	-0.132	0.389				
	P	**0.001**	0.624	**0.005**	**0.038**	0.135	0.607	0.347	0.133	0.056	0.237	0.072	**0.032**	0.339	0.300	0.868	0.315	0.221	0.408	0.024	0.230	0.134	0.022	0.721	0.241	0.932	**0.002**				
pH	*r*	0.213	-0.117	0.169	0.021	0.315	-0.105	0.229	0.274	0.095	0.037	0.093	0.106	0.280	0.292	0.359	0.207	-0.028	0.244	0.194	0.270	0.177	-0.008	-0.071	0.204	-0.113	0.274	0.050			
	P	**0.042**	0.970	**0.047**	0.381	**0.009**	0.816	**0.020**	**0.014**	0.193	0.314	0.183	0.194	**0.009**	**0.007**	**0.005**	**0.028**	0.441	**0.018**	0.055	**0.008**	**0.054**	0.361	0.771	**0.031**	0.866	**0.016**	0.285			
Longitude	*r*	0.126	0.164	0.213	0.420	0.098	0.139	0.304	0.263	-0.007	0.018	-0.011	-0.050	0.207	0.258	0.155	0.164	0.069	0.228	0.303	0.281	0.191	0.086	0.101	0.156	0.029	0.199	0.097	0.000		
	P	**0.011**	**0.006**	**0.001**	**0.001**	**0.034**	**0.011**	**0.001**	**0.002**	0.560	0.369	0.547	0.805	**0.001**	**0.001**	**0.007**	**0.005**	0.087	**0.001**	**0.001**	**0.001**	**0.001**	**0.027**	**0.036**	**0.002**	0.349	**0.001**	**0.035**	0.488		
Latitude	*r*	0.183	0.114	0.328	0.421	0.097	-0.018	0.407	0.348	-0.008	-0.020	-0.006	-0.053	0.248	0.321	0.010	0.217	0.106	0.315	0.402	0.392	0.287	0.087	0.051	0.217	-0.069	0.315	0.193	-0.036	0.853	
	P	**0.012**	**0.050**	**0.001**	**0.001**	0.092	0.557	**0.001**	**0.001**	0.444	0.556	0.466	0.706	**0.003**	**0.001**	0.366	**0.005**	0.120	**0.001**	**0.001**	**0.001**	**0.002**	0.185	0.171	**0.006**	0.774	**0.001**	**0.006**	0.690	**0.001**	
Moisture	*r*	-0.011	0.174	0.140	0.003	0.070	0.002	0.119	0.135	0.184	0.124	0.256	0.160	0.089	0.089	-0.064	0.104	-0.064	0.080	0.098	0.081	0.036	-0.006	-0.005	0.138	0.149	0.157	0.067	-0.015	0.009	0.048
	P	0.486	**0.015**	**0.043**	0.455	0.22	0.424	0.083	**0.08**	0.057	0.097	**0.005**	0.065	0.149	0.141	0.743	0.119	0.709	0.164	0.153	0.161	0.317	0.454	0.467	0.057	0.102	**0.042**	0.208	0.541	0.375	0.179

Note: r, and p is the correlation coefficient and significance value, respectively; TCE represents the total content of all the elements.

Table S3 Summary of the sequence data.

Samples	Prokaryotic community				Fungal community			
	Valid reads	Average length (bp)	Normalized reads	OTUs	Valid reads	Average length (bp)	Normalized reads	OTUs
G1	32324	380.04	30126	229	34022	239.91	30198	79
G2	38462	379.92	30126	234	30276	244.26	30198	72
G3	44451	380.36	30126	235	32866	243.67	30198	141
G4	39148	378.33	30126	700	42181	242.04	30198	33
G5	41799	376.36	30126	438	37173	260.58	30198	22
G6	38776	375.89	30126	139	31028	235.25	30198	16
G7	36319	375.9	30126	189	41866	248.55	30198	33
G8	40973	375.92	30126	220	37209	263.5	30198	15
G9	43803	376.17	30126	515	31541	300.33	30198	24
GT1	34489	377.14	30126	1761	35031	236.16	30198	164
GT2	43336	377.26	30126	1742	39717	240.77	30198	89
GT3	34992	377.29	30126	1816	41907	237.43	30198	176
W11	32450	376.09	30126	1953	36846	242.62	30198	47
W12	34600	376.26	30126	2117	32980	251.82	30198	95
W13	31517	376.15	30126	1876	30457	234.37	30198	112
W21	34260	376.25	30126	1123	34057	267.08	30198	25
W22	40511	376.31	30126	1258	32998	259.52	30198	64
W23	34549	376.19	30126	2394	40358	241.37	30198	48
W31	44700	376.22	30126	2376	41203	246.05	30198	71
W32	41909	376.33	30126	2166	32427	309.99	30198	39
W33	40178	376.3	30126	2082	43397	215.78	30198	97
WT1	41130	377.11	30126	1893	36878	244.55	30198	338
WT2	42020	377.06	30126	1947	44771	239.84	30198	366

WT3	40754	376.97	30126	1929	30642	242.52	30198	214
Y11	43843	375.92	30126	137	35665	249.8	30198	27
Y12	44161	375.86	30126	77	39829	289.02	30198	12
Y13	34067	375.69	30126	889	44079	269.02	30198	35
Y21	40854	375.88	30126	82	32497	233.66	30198	18
Y22	38243	375.9	30126	74	43699	244.43	30198	16
Y23	38871	375.92	30126	130	30651	233.26	30198	15
Y31	43692	375.85	30126	2006	30669	262.03	30198	87
Y32	38488	376.11	30126	1010	34053	247.28	30198	26
Y33	35545	375.9	30126	1170	30416	251.69	30198	33
YT1	37272	376.51	30126	2289	39126	261.4	30198	307
YT2	37246	376.85	30126	2118	37584	249.97	30198	54
YT3	44379	376.84	30126	2265	31776	246.41	30198	172

Table S4 Alpha-diversity indices of the microbial communities. Chao1: the Chao1 estimator; Shannon: the Shannon index.

Sample	Prokaryotic community		Fungal community	
	Chao1	Shannon	Chao1	Shannon
G1	285.9	5.33	80	5.48
G2	307.3	5.26	73.5	5.39
G3	303.9	5.13	140	5.77
G4	760.8	6.87	33	4.25
G5	471.1	3.8	22	4
G6	146	2.83	16	3.45
G7	205.7	2.61	33	4.34
G8	230	3.36	15	3.11
G9	601.3	5.45	27	0.73
GT1	2046.6	8.87	163.2	5.66
GT2	1971.6	8.87	89	4.35
GT3	2225.5	8.9	178.5	5.63
W11	142	2.39	30	3.82
W12	86	2.2	18	1.07
W13	1016.7	7.31	63	3.52
W21	82.8	2.29	19.5	2.26
W22	75.3	2.11	16.3	2.97
W23	136	2.55	16	0.88
W31	2609	8.43	99	4.26
W32	1092.5	6.29	26.3	3.5
W33	1329.9	7.04	36.3	3.7
WT1	2848.9	9.26	316	5.51
WT2	2683.2	9.22	54	4.03

WT3	2785	9.45		175.1	4.3
Y11	2521.4	8.12		47	3.76
Y12	2719.8	8.41		100	4.01
Y13	2600.8	7.77		114.6	2.64
Y21	1367.7	7.66		73.2	2.07
Y22	1160.7	7.58		32	0.07
Y23	3035.7	9.02		48.3	4.07
Y31	3287.3	8.57		71.3	3.16
Y32	2621	8.49		42	0.71
Y33	2804.2	8.15		97	1.74
YT1	2190.4	9.02		349.1	5.91
YT2	2251.1	9.17		376.3	5.43
YT3	2360.8	9.14		235.4	4.49
